# Discourses mapped by Q-method show governance constraints motivate landscape approaches in Indonesia

**DOI:** 10.1371/journal.pone.0211221

**Published:** 2019-01-31

**Authors:** James Douglas Langston, Rowan McIntyre, Keith Falconer, Terry Sunderland, Meine van Noordwijk, Agni Klintuni Boedhihartono

**Affiliations:** 1 Centre for Tropical Environmental and Sustainability Science, College of Science and Engineering, James Cook University, Cairns, Queensland, Australia; 2 Tanah Air Beta, Batu Karu, Tabanan, Bali, Indonesia; 3 Faculty of Forestry, Forest Sciences Centre, University of British Columbia, Vancouver, Canada; 4 World Agroforestry Centre, ICRAF Southeast Asia Regional Office, Bogor, Indonesia; 5 Plant Production Systems, Wageningen University and Research, Wageningen, the Netherlands; University of Maryland Baltimore County, UNITED STATES

## Abstract

Interpreting discourses among implementers of what is termed a “landscape approach” enables us to learn from their experience to improve conservation and development outcomes. We use Q-methodology to explore the perspectives of a group of experts in the landscape approach, both from academic and implementation fields, on what hinderances are in place to the realisation of achieving sustainable landscape management in Indonesia. The results show that, at a generic level, “corruption” and “lack of transparency and accountability” rank as the greatest constraints on landscape functionality. Biophysical factors, such as topography and climate change, rank as the least constraining factors. When participants considered a landscape with which they were most familiar, the results changed: the rapid change of regulations, limited local human capacity and inaccessible data on economic risks increased, while the inadequacy of democratic institutions, “overlapping laws” and “corruption” decreased. The difference indicates some fine-tuning of generic perceptions to the local context and may also reflect different views on what is achievable for landscape approach practitioners. Overall, approximately 55% of variance is accounted for by five discourse factors for each trial. Four overlapped and two discourses were discrete enough to merit different discourse labels. We labelled the discourses (1) social exclusionists, (2) state view, (3) community view, (4) integrationists, (5) democrats, and (6) neoliberals. Each discourse contains elements actionable at the landscape scale, as well as exogenous issues that originate at national and global scales. Actionable elements that could contribute to improving governance included trust building, clarified resource rights and responsibilities, and inclusive representation in management. The landscape sustainability discourses studied here suggests that landscape approach “learners” must focus on ways to remedy poor governance if they are to achieve sustainability and multi-functionality.

## Introduction

Landscape scale interventions to achieve economic development while supporting environmental integrity are being promoted in Indonesia as a means to achieve the Sustainable Development Goals [1]. Commonly referred to as ‘landscape approaches’, these interventions are used in intergovernmental initiatives and by governments, by research and academic institutions, NGOs, as well as the private and business sectors [2]. Such space-based approaches are considered preferable to ‘commodity’ based approaches to managing the environmental, social, and economic sustainability of global production systems [3–5]. The attraction of landscape approaches is the perceived potential for delivering conservation and development synergies and minimizing trade-offs [6]. Landscape scales are considered by many to be where broader sustainability challenges are most manageable [7]. Recent discourse has suggested that the global sustainable development community might coordinate to unlock ‘potentially trillions’ of dollars to be directed into landscape approaches for achieving sustainable development [8]. Such approaches are, of course, compelling and have permeated almost all corners of the development and conservation discourse. Yet, in reality, long-term and sufficient funding for the conservation of natural resources and economic development of rural societies remains elusive.

Common conceptions of landscape approaches cover a substantial diversity of actions, applied in a range of contexts [1]. To-date there remains a lack of consensus on what a landscape approach really entails [9]. There is, as yet, no widely accepted definition of a landscape approach, primarily because some landscape approach theorists maintain that there should not be a singular rigid definition as these sorts of integrated geographically defined approaches have to be used in a diversity of contexts [10]. Broadly, a landscape approach can be considered “a long-term collaborative process bringing together diverse stakeholders aiming to achieve a balance between multiple and sometimes conflicting objectives in a landscape or seascape” [2]. Landscape approaches adhere to a set of principles that are meant to steer the governance of landscapes to better reconcile and integrate conservation and development efforts [11]. They involve integrating land management with aim of enhancing social and environmental outcomes for the sake of sustainable and inclusive development [12]. While landscapes, considered as social-ecological systems, are the entry unit for analysis and implementation, landscape approach principles explicitly consider multi-scalar interactions and outcomes. Landscapes are not delimited by environmental variables such as watersheds, or political variables such as jurisdictions, rather by a combination of social and environmental determinants. There is a growing body of literature exploring the origins, history, and evolution of landscape approaches [10, 13–17], but some are concerned that a lingering ‘conceptual capaciousness’ means that the majority of integrated approaches, and most environmental governance, can resemble the landscape approach, therefore detracting from its meaning [18]. While a set of principles and guidelines [11] and generic theories of changes [2, 19] for landscape approaches have been developed, more rigorous conceptual and analytical frameworks are largely missing [18]. Due to the scope and transdisciplinary nature of landscape approaches, there remains a wide range of terminology and ontological divergences on how landscape approaches are applied in practice [13].

Landscape approaches are not immune from critique. Some are concerned with unrealistic claims of win-win goals [20] and the difficulty in their application [21], while some claim landscape approaches are being used to de-politicize the problems apparent in social-ecological systems and entrench neoliberal exclusionary development [22, 23]. The pre-conditions for successful landscape approaches are indeed daunting [24], but there are growing interests in ways to co-generate knowledge and policy to redress inequalities. Some of those tools are discussed elsewhere [25, 26].

Knowledge is often contested between multiple actors in complex landscapes [27, 28]. If implementers of landscape approaches are going to succeed in achieving their goals, they must come to terms with the actors and discourses at multiple scales; problem framing must be rigorous and collaborative [29]. This is because the challenges of social-ecological systems are complex and often stem from poorly coordinated decisions, where different elements of society frame problems in terms of their own needs and aspirations, leading to unsatisfactory, and often conflicting, zero sum outcomes [7, 30, 31].

A recent review shows the prevalence of the use of landscape approaches in South and Southeast Asia [6]. In Indonesia, investments that are driving change [32], are sought to be governed by landscape approaches [4, 30, 33, 34]. Indonesia has adopted broader landscape approaches in the implementation of projects to Reduce Emissions from Deforestation and Forest Degradation (REDD+), ecosystem restoration concessions, and forestry management units (Kesatuan Pengelolaan Hutan or KPH). The largest estate crop companies are moving towards the implementation of landscape approaches as part of their sustainability strategies [35]. But studies have shown that landscape governance does not usually come from formally planned legislation, rather through “institutional bricolage”, where diverse actors create new institutional space by creatively combining local institutions with externally introduced mechanisms, constructing hybrid institutions adapted to landscapes social-ecological contexts [28, 36]. Consequently, landscape approaches resemble ‘muddling through’ [37], as implementers realize grand designs fail to deliver satisfactory sustainability outcomes [38]. They should ‘not be seen as prescriptive approaches to spatial planning’ [2].

In Indonesia, the challenges for sustainable and inclusive development are writ large. The country contains the world’s second largest tropical rainforest, and the most extensive and most biodiverse marine ecosystems [39, 40]. It is also home to the world’s fourth largest, rapidly growing, and culturally diverse population, who are pursuing economic well-being [41, 42]. Indonesia’s governance arrangements are notoriously complex and dynamic; rapidly changing legislation and shifting hierarchies of control have beset the stewardship of natural, economic, and social assets with difficulties [30, 43–45]. Many development benefits have often accrued inequitably, especially where large investments drive landscape scale change [46, 47]. Indonesia’s development threats and opportunities, alongside their rich but degrading nature demand governance that can deliver optimal outcomes for people and nature [4, 48]. An in-depth discussion of the sustainability discourses in Indonesia is beyond the scope of this paper because our primary goal is to use a relatively objective method to illuminate the issues of landscape sustainability according to landscape sustainability experts who, we hypothesize, all have their own interpretations of the context of sustainability in Indonesia.

The vast array of different and contextualized social-ecological conditions in Indonesia means there are now a variety of diverse applications of the already conceptually vague landscape approach [18]. The broad range of understandings means that even within a single landscape, implementers are likely to diverge in their perspectives as to what the obstacles are for landscape sustainability. Rather than become a discursive barrier, different perspectives can be made transparent, and if management coalitions account for them, they can enable more equitable delivery of benefits to a broad range of actors within a landscape. As a transdisciplinary team attempting to influence development outcomes in Indonesia, the authors and participants of the study are inspired by this diversity to achieve greater understanding on what the obstacles are for landscapes if we are to influence and understand their development trajectories.

Considering what is at stake in Indonesia’s landscapes both for people and their environment, the sustainability challenges deserve greater attention: what are the problems, and according to whom? Opportunities to learn from the existing set of circumstances as well as the diversity of approaches depend on how we interpret the variation among discourses of those involved in landscape approaches in the field. Q-methodology [49] has shown potential for uncovering underlying narratives of sustainability, resource management, and development issues, wherein power and politics drive decisions [50–52]. The method combines unique qualitative and quantitative research principles [53]. Q is particularly suitable for studying highly debated and contentious phenomena, such as landscape approaches, because it aims to identify different or shared ways of thinking on a topic, keeping the researcher’s perspective relatively independent from the procedure and results [54].

Clear evidence enables systemic learning [55] and defining stakeholder perspectives can be useful for both knowledge brokers and boundary institutions [27] aiming to influence or induce change in complex and contested landscapes [56–58]. Articulating the full range of stakeholder perceptions supports legitimacy and buy-in to any intervention aimed at solving problems affecting social-ecological systems [59]. Clarity of points-of-view is critical in the complexity and ambiguity caused by de-and re-centralization of governance arrangements such as in Indonesia [60]. Indonesia’s knowledge brokers and boundary institutions would then be more able to leverage points of consensus and address controversies, fundamental to building the trust necessary for reconciling the trade-offs inherent in integrated landscape initiatives [7, 20]. Zabala, Sandbrook [61]show that applying Q-methodology uniquely allows identification of the range of nuanced perspectives in a structured way. Furthermore, Q helps identify divergence and consensus around key topics, which can then be used to facilitate critical reflection among actors and assess management strategies.

This paper heeds a call by Opdam [62] for scientific methods to better interact with social processes, to bridge the gap between science and practice by grappling with underlying narratives of landscape sustainability. During a gathering of landscape approach practitioners and associated academics we explored perspectives on what prevents landscapes in Indonesia from functioning as well as they could. Functionality was not considered by the group to be an endpoint [63]. Functionality was conceived to mean improved sustainability—delivering multiple goods and services to satisfy the full range of actors in an equitable and accountable way. Functionality was not defined according to normative or concrete criteria, rather the goal was to explore the full range of the participants internal understandings of sustainability, and how sustainability is constrained in ‘places’[64]. Through our discourse analysis we identified points of divergence and consensus over core concepts and we identify vantage points people have when using landscape approach principles in their work or research. Our results contribute to more comprehensive narratives on what motivates the implementation of landscape approaches, reducing the ambiguity surrounding landscape-scale sustainability in Indonesia. We conclude that to effectively coordinate landscape interventions for achieving impact, investments must contribute to rigorously transparent evidence-based problem framing. Management coalitions that allocate resources must understand where peoples’ values intersect politically, and they must be accountable to their own divergent political vantage points when seeking to remedy inadequate governance.

## Methods

### Setting

In September 2017, during a gathering of landscape approach practitioners and associated academics at a ‘Learning Landscapes’ retreat in Indonesia, we took the opportunity to explore the perspectives on what prevents landscapes in Indonesia from functioning as well as they could, as discussed above. The objective of the retreat was to bring together leaders of landscape and seascape initiatives in Indonesia for them to compare approaches, challenges and achievements. The retreat was held in Setulang Village, Malinau District, North Kalimantan, Indonesia. Malinau district was the location of a major initiative by CIFOR from 1994–2009 to develop integrated landscape approaches to the understanding of large-scale forest transformation processes [65]. Five people who worked on the initiative at that time were present at the retreat. Information and publications from that period were available to the retreat participants; 34 participants were present for all the activities. The idea to perform a Q methodology arose during the retreat, it was not pre-planned. There was consensus among the participants that exploring the potentially wide-ranging views among the retreat attendees would stimulate debate and would lead to a more rewarding ‘learning landscapes’ retreat. The specific methodology was proposed to the group and all attendees gave consent verbally and were enthusiastic to participate in the exercise. Ethics approval for this study was obtained from the James Cook University Ethics Committee (Ethics Approval Application ID H4756).

### Q method

Q-methodology provides a comprehensive approach to the study of perspectives and subjectivity [49, 66, 67]. It uses abductive reasoning to understand the viewpoints and the differences in a sample population. Abductive logic, as opposed to deductive or inductive logic, involves seeking the most likely explanation from an acknowledged incomplete set of observations. Q-methodology can reveal complexity of values and perspectives that are not obtained by standard surveys. For these reasons it has been used to study other development, sustainability, and natural resource issues [49, 54]. Our three main steps in applying the Q-method were: 1) developing a concourse, 2) obtaining Q-sorts of these statements from 34 participants, 3) analysing the data for overall level of agreement, and for recognizing distinguishable discourses with a principal component analysis.

**Fig 1:**
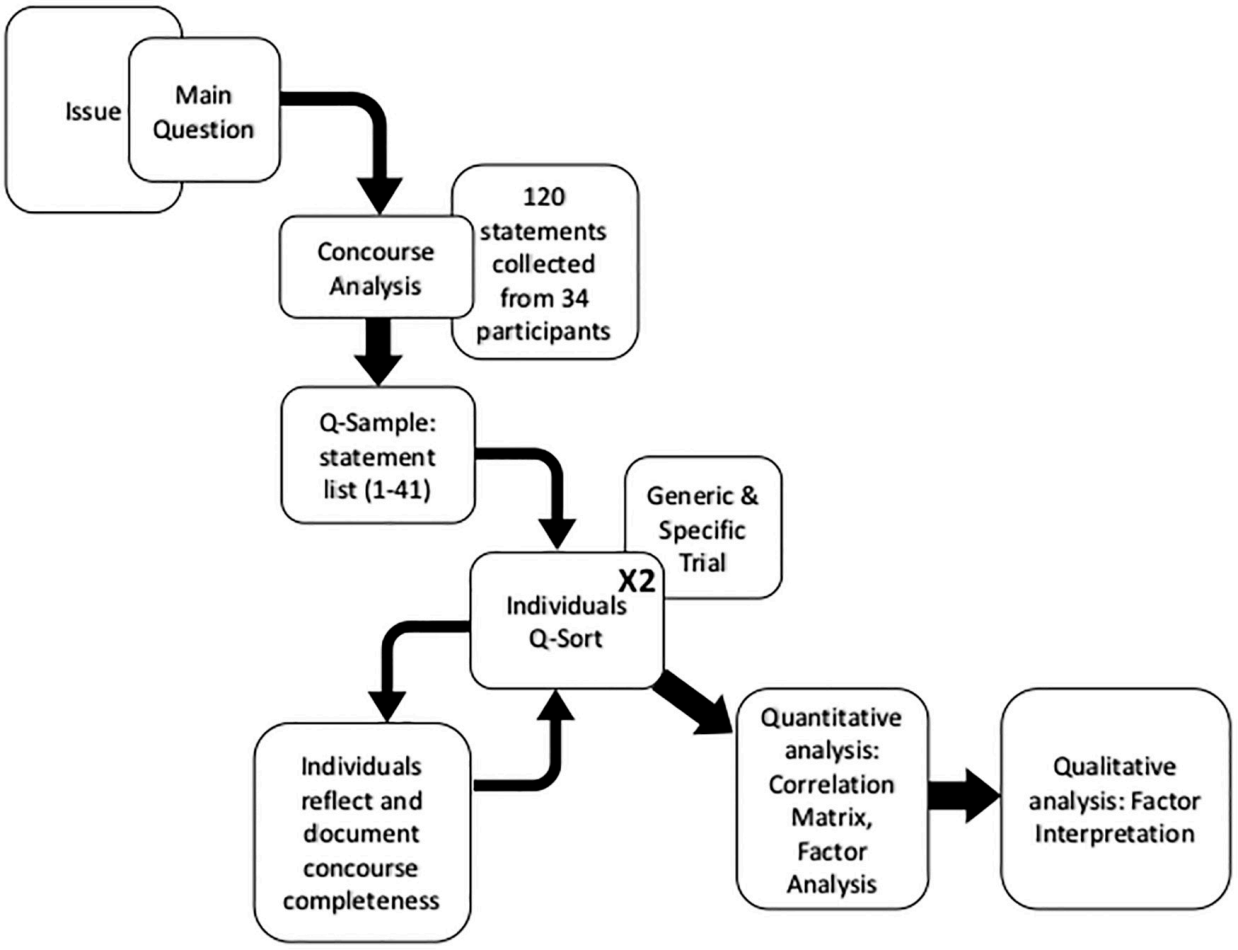
Q methodology flow diagram for our study.

Fig 1 describes the process of our Q method application. The overall issue was the overarching theme of the retreat: landscape approach challenges and achievements. We determined the Q-question “what prevents landscapes in Indonesia from functioning as well as they could?” out of consideration for the pertinent question for practitioners: what hinders change in the landscapes that we are trying to influence onto trajectories of sustainability?

To develop a concourse, we collected statements from all of the 34 participants (P-set). Participants formed statements on the basis of their knowledge of the impediments to achieving landscape level sustainability in Indonesia, whether through experienced project implementation or independent observation. The statements were in response to a question: “what prevents landscapes in Indonesia from functioning as well as they could?” Participants could suggest as many statements or phrases as they deemed sufficient to capture the range of issues that were important. In this case the participants listed 120 phrases in total. Concourse analysis [66] seeks to capture the full breadth of discussions related to the issue, the results of which becomes the raw material for the Q-sample (the set of questions used for the Q-sorting). We deemed the 120 statements sufficiently diverse, capturing the full breadth of the potential factors influencing landscape functionality. We distilled the 120 statements to 41, our final Q-sample (Fig 1). We reduced the statements by combining similar statements into themes and then combined aspects of similar statements to reduce specific overlaps. When eliminating specific statements, we did so in a way that minimized the loss of the diversity of ideas from the original set of themes.

Our P-set (n = 34, 25 males) was comprised of a variety of academics and practitioners, with some straddling both domains. We selected the 34 participants to capture a wide diversity of (1) sectors of society, (2) reasons for implementing landscape approaches, and (3) degrees of practitioner and academic involvement. All participants were familiar with the theory of landscape approaches [11], and the challenges of their application [2] ahead of the retreat. The majority of the participants had been involved in the application of landscape approaches in either one or many initiatives, within Indonesia or globally. All were familiar with the Indonesian context through their knowledge of CIFOR’s Malinau research forest in the 1990s-2000s and from many other Indonesian case studies. Twenty-one participants were applying their own landscape approach in Indonesia at the time of the retreat. Nineteen participants were Indonesian, fifteen were international including: five Australian, one British, one Dutch, one French, one German, one Irish, one Russian, one Spanish, one Vietnamese, and two from the United States of America. The participants represented different sectors of society: eighteen from academia, two from the private sector, and ten from various NGOs. Four reported straddling both NGOs and academia, and four reported holding civil servant positions while studying in academia. The academics were comprised of Masters’ students n = 7, PhD students n = 3, and professors and lecturers n = 8. All students and academics come from development practitioner-based backgrounds. The academics were all applied scientists also working in civil society organizations or private sector companies aiming to steer development trajectories in tropical landscapes. The students were all practitioners enrolled in a ‘practice-based’ development program with the aim of influencing development from a broad-based, multi-disciplinary foundation. Civil servants represented central government positions in Vietnam and Indonesia, and district level governments in Indonesia.

**Fig 2:**
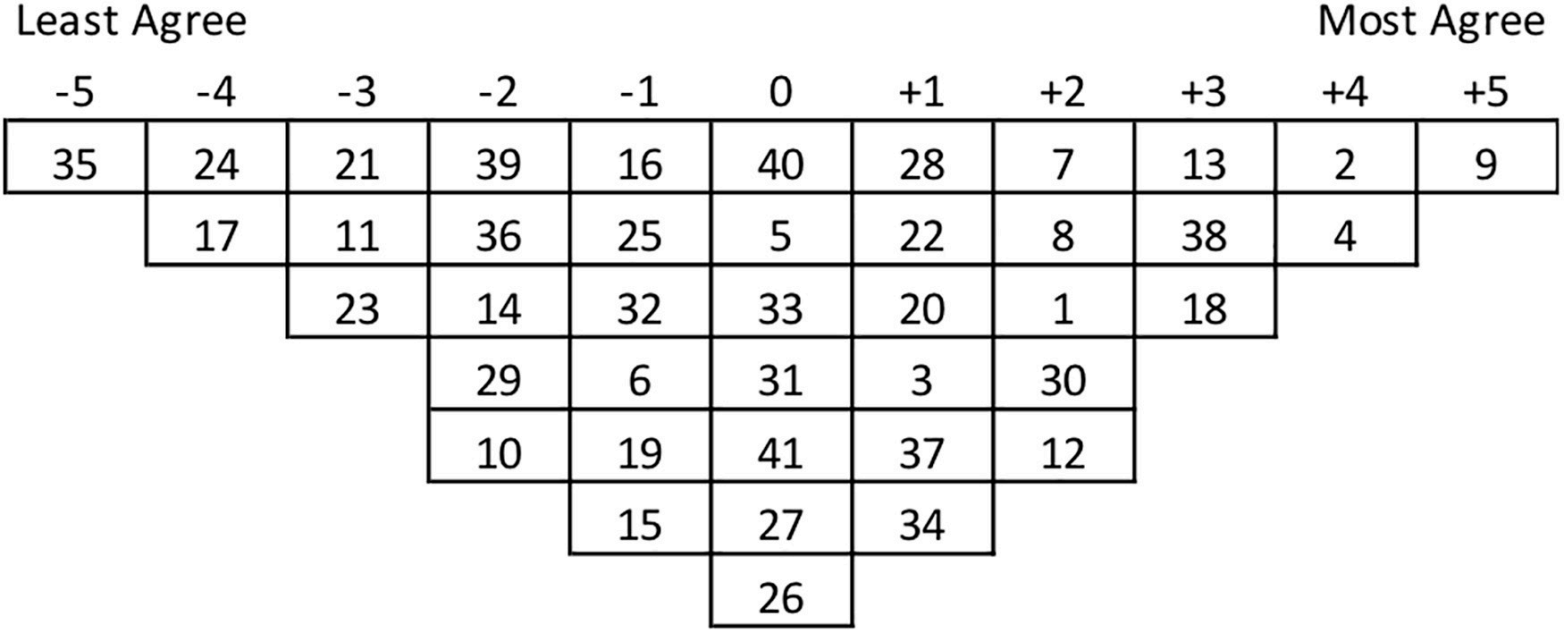
Sample of a Q-Sort. The chart forces a normal distribution for the 41 statements. Each participant must allocate every statement into a box. The numbers in this example represent the statements 1–41 (Fig 3). The position of each statement indicates the level of agreement.

To obtain Q-sorts from the P-set, each participant took all 41 statements written on square pieces of paper and initially classified them as ‘agree’, ‘neutral’ or ‘disagree’. Then they placed all 41 statements onto a Q-sort board (see design in Fig 2). Each statement was assigned a number 1–41 and we recorded each participant’s final sort, an example of which is shown below in Fig 2. Opportunity to reflect on the 41 statements was provided to each participant after their Q-sort; we asked and documented if there was anything missing from the Q-sample or whether it reflected the comprehensive concourse. All participants were asked to Q-sort twice. Once generally for landscapes in Indonesia, and a second time for a specific landscape they were either familiar with or where they were working. The premise of sorting twice was to interrogate the degree to which participants perceive differences imposed by local context or whether they consider that they can apply a predetermined set of generic concepts applicable broadly.

### Analysis

We used an open-source software, Ken-Q Analysis (https://shawnbanasick.github.io/ken-q-analysis/#section1 Version 0.11.0), to build the correlation matrix, perform the principle component analysis, and flag the defining sorts. We ran the analysis separately for the generic trial and the specific trial. Similarities between Q-sorts are identified from the correlation matrix and the principle component analysis classifies information based on the correlations between Q-sorts. We extracted eight principal components and applied a varimax rotation to the first five factors, which cumulatively explained 54% of the variation for the generic trial, and 55% for the specific trial. Choosing how many factors to keep for rotation is based on how many factors are significantly distinct. There needs to be enough factors to represent the sorts of groups represented in the P-set. Each Q-factor is the average perception of respondents with similar views. However, there are no fixed rules for determining how many factors to keep for analysis. Deciding how many factors to keep is a mix of subjectivity and objectivity; “scientists should not make a decision based on statistical rules only, but also use qualitative knowledge of the context” [51]. The results of keeping five factors divided the discourses into sufficiently comprehensible nuanced similarities and differences between groups. Keeping five factors also divided the p-set into intuitively distinct groups of people with Indonesian, international, disciplinary, and workplace backgrounds. The cumulative variance within the P-set at over 50% is used as an acceptable determinant elsewhere [51, 52], so we deemed a 55% variance for both trials using five discourse factors to be most appropriate.

After establishing the discourse factors for the generic trial and specific trial, we examined the distinguishing statements at the ‘most agree’ and ‘least agree’ sections of the Q-sort (refer to S1 Table). We derived independent names for the factors and looked for overlaps or differences between the generic and specific trial discourse factors, based on the characteristics of the statements. We labelled the first four discourse factors for the generic and specific trials the same due to their similarity (i.e. discourse factor one in the generic trial received the same name as discourse factor one in the specific trial). One factor in the general trial and one in the specific trial, diverged enough to merit different labels—factor five in the general trial, and factor five in the specific trial.

Defining statements from the Q-sample that were distinguishable from each factor were flagged, significant at p < 0.05, according to the standard Q criteria, which includes minimizing confounding factors (p < 0.05 labelled in S1 Table, with D, with p < 0.01 labelled D*). The P-set is divided up by Q-sort responses closest to each other and a model Q-sort is created for each factor from the results of the factor loading of the flagged Q-sorts. Out of 34 Q-sorts, nine Q-sorts remain without a significant loading—they did not belong to any specific discourse but shared opinions of all other respondents. Factors, henceforth referred to as discourse factors, were interpreted based on the correlation matrix that converted the flagged average of each person’s score for each statement to a normalized factor score (or Z-score) to standardize the distribution across the statements.

The following results describe discourses stemming from the Q-sort sample. The narrative which emerges represents their collective experiences and interactions with conservation and development processes. They are not representative of local people living in the landscapes of concern, but that does not discount their solidarity for local people, their interests and their environments. Respondents are a subset of ‘landscape approach’ experts who have an interest in steering the trajectory of development in tropical landscapes. The limits of the study are therefore bound by the histories and personal perspectives of the participants.

## Results

Overall, statements referring to “corruption” and “lack of transparency” scored highest, and statements on agricultural policies and biophysical factors such as topography and climate change, the lowest. When participants considered a landscape they knew best, the results changed slightly: the rapid change of regulations, limited local human capacity and inaccessible data on economic risks increased in relevance, while scores for inadequacy of democratic institutions, overlapping laws and corruption became less important. Both generic trial and specific trial highlight that corruption, lack of accountability, policy and sectoral inconsistencies, weak enforcement of rules and regulations, divergent goals and unsatisfactory stakeholder respect are ranked as the main constraints to landscape functionality. Table 1 shows a list of the most and least constraining factors according to our P-set for both the generic and landscape specific trials. The most illustrative set of main constraints and least constraints fell at a convenient Z-score threshold plus one and minus two (see Table 1).

**Table 1:** Overall results for biggest and least constraining factors that prevent landscape functionality in Indonesia.

	Degree of constraint	Statement
General trial	Main constraints (Z-score > 1)	Corruption, personal benefits for those issuing permits
		Lack of accountability to civil society, opaque decision making, lack of transparency
		Inconsistencies between sectoral policies and misalignment of government structures
		Weak enforcement of existing regulations, poor monitoring of actual change
		Lack of a common (negotiated, agreed) goal for the landscape as a whole
		Unclear and contested tenure rights, conflicting claims
		Differing goals of stakeholders in the landscape, lack of recognition and respect for various perspectives and interests
		Exclusion or underrepresentation of important stakeholders in decision making
	Least important (Z-score < -2)	Topography constraints to transport, durable roads
		Global climate change, locally changing rainfall patterns
		Rice focus of agricultural policies
Specific trial	Main constraints (Z-score > 1)	Inconsistencies between sectoral policies and misalignment of government structures
		Lack of accountability to civil society, opaque decision making, lack of transparency
		Corruption, personal benefits for those issuing permits
		Weak enforcement of existing regulations, poor monitoring of actual change
		Lack of a common (negotiated, agreed) goal for the landscape as a whole
		Differing goals of stakeholders in the landscape, lack of recognition and respect for various perspectives and interests
		Increased pressure on land and resources leads to government priorities for economic growth over environmental integrity
		Unequal bargaining power, large-scale concessions without local consent
	Least important (Z-score < -2)	Topography constraints to transport, durable roads
		Rice focus of agricultural policies
Variation between general and specific trial	More influential when referring to own landscape (highest positive change)	Regulations change too quickly to be fully applied
		Limited human capacity (knowledge, decision making) within communities and government
		Lack of economic data on risk, price fluctuations, market dynamics
		Slow transition from subsistence focus to active participation in wider economic activities (tie for 3rd)
	Lower influence when referring to own landscape (highest negative change)	Inadequate democratic processes and institutions
		Corruption, personal benefits for those issuing permits
		Overlapping partly contradictory laws with loopholes and lack of grievance procedures

### Discourse analysis

**Fig 3:**
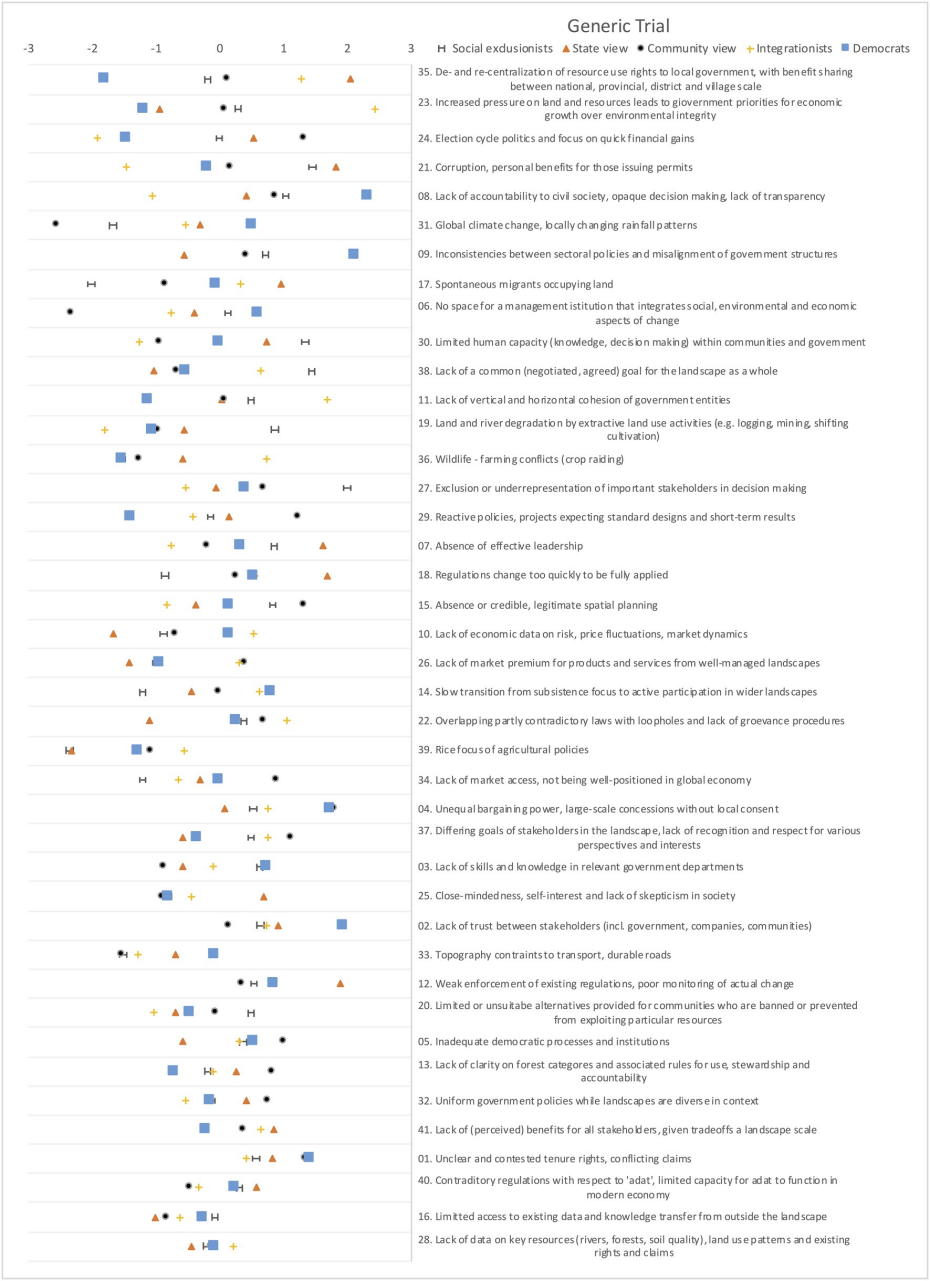
Q-statements and their z-scores for the general trial. Ordered from most distinctive at the top to most consensus at the bottom (based on z-score differences). Distinguishing statements that defined each discourse are found in S1 Table of the supplementary material.

**Fig 4:**
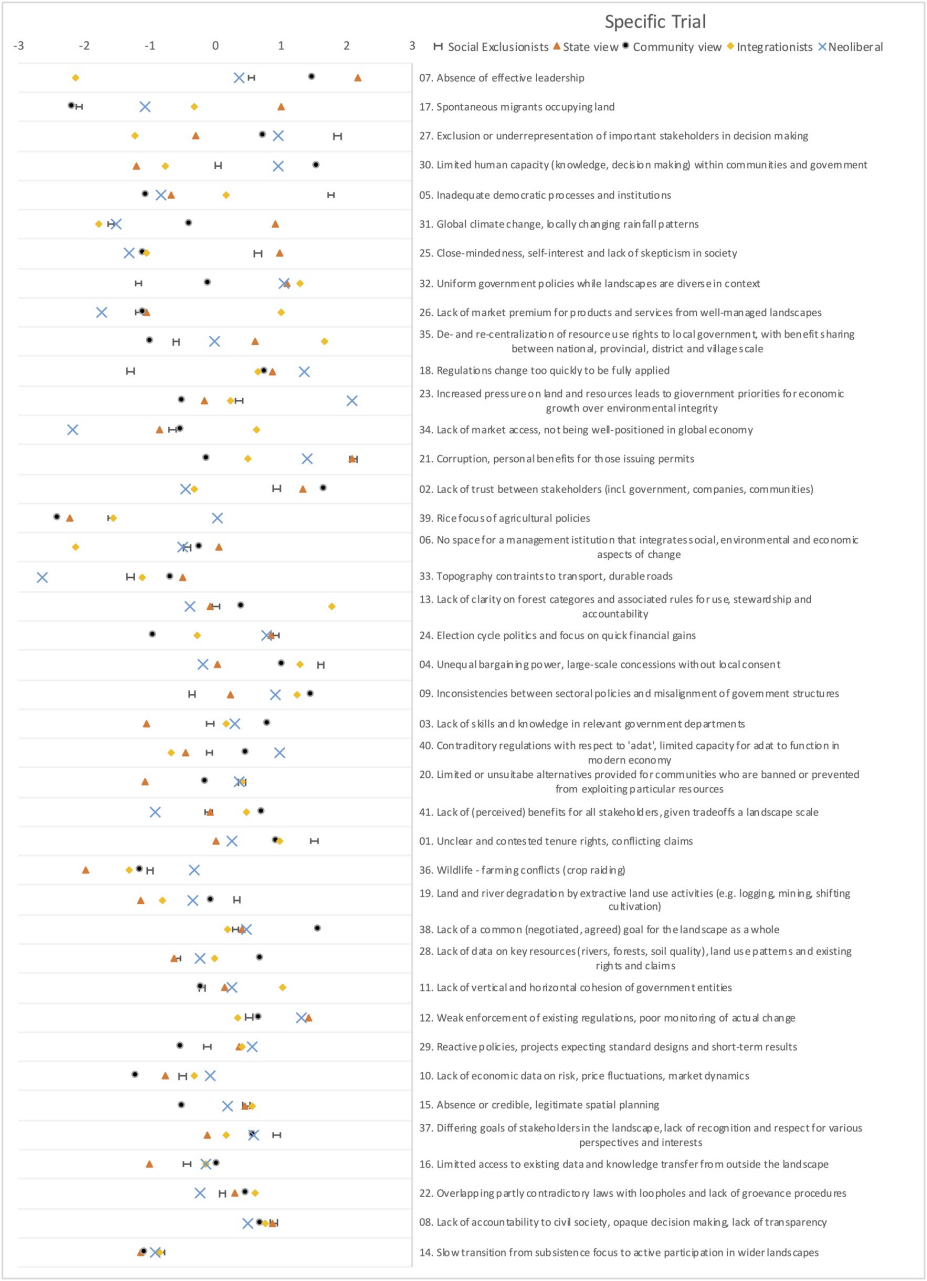
Q-statements and their z-scores for the landscape specific trial. Ordered from most distinctive at the top to most consensus at the bottom (based on z-score differences). Distinguishing statements that defined each discourse are found in S1 Table of the supplementary material.

Five discourse factors explain 54% of the variance for the general trial and five explain 55% of the variance for the specific trial. Based on our review of the thematic elements among the distinguishing factors on the ‘most agree’ and ‘least agree’ end of the Q-sort discourse factors (see S1 Table), we distinguished six total discourses with the following titles: (1) social exclusionists, (2) state view, (3) community view, (4) integrationists, (5) democrats, and (6) neoliberals. We determined the titles of the discourses from the emergent properties of the ‘most’ and ‘least’ constraining statements of each factor. While the general trial and the specific trial both produced five factors for a total of ten factors, eight of them paired. These eight paired as four in each trial because they resembled each other enough to merit the same discourse title. That means one factor in each trial merited different names; the ‘democrats’ and ‘neoliberals’ were unique to the generic trial and specific trial, respectively. The first four factors should be considered as discourses with slightly resituated perspectives from general to specific trials. The supplementary material contains a table (S1 Table) comprised of the most and least constraining statements for each discourse factor, in addition to the rankings of each statement for each discourse factor (S2 and S3 Tables). Figs 3 and 4 show the discourses for the general trial and landscape specific trial respectively, by showing the degree to which statements distinguish from each other at the top, to the degree of consensus at the bottom.

#### Group 1. Social exclusionists

The first discourse group perceives the main hindrances to landscapes functionality as a function of the exclusionary nature of development. Immigration is not perceived as an issue due to ideals of inclusive development. Rather, the variety of actors are under the imposition of predatory institutions involved in corruption, patronage, and powerful extractive groups that contribute to a mode of ‘accumulation by dispossession’ [68]. The group is concerned that decision-makers are not comprised of the full range of people in the landscape and that the decision-making apparatus excludes people (local communities) even though they will affect landscape outcomes. The unimportance of slow livelihood transitions might reflect ideals of rights to self-determination for the people in the landscape, regardless of their origins. The majority of the respondents in this discourse are not Indonesian and have backgrounds in anthropology or on ‘people-centric’ approaches to development, such as in NGOs working on human rights and conflict resolution. Global and local climate concerns do not concern this group, likely due to the perception that it is fundamentally something people must adapt to, and will adapt to, if they are given equal access to development opportunities. In the specific trial, the constraints distil to basic tenets of democratic process for people, while discounting the policy and regulatory environment. This group sees everyone as deserving a fair chance, supported by institutions with integrity.

#### Group 2. State view

Perceptions of respondents that ‘see like a State’ [69] are related to aspects of effective oversight of legislation, regulation, enforcement, and leadership. Noticeably, immigration is a problem for landscape functionality—this was apparent when participants thought of a specific landscape. The factors that do not hinder landscape functionality are related to knowledge, human capacity, and insufficient freedom of choice for communities. This makes sense if the problems are a matter of executing and following orders. In the landscape specific trial, the main concern over unclear government authority from years of de-and re-centralization disappears [70, 71]. As the participants focused on their landscape, the context of complicated resource use-rights became less prominent, and executive assertions became more prominently actionable. This group was represented primarily by Indonesian nationals who have worked for natural resource management/conservation organizations in multiple areas across Indonesia.

#### Group 3. Community view

A community development theme runs through the third discourse group. The main hindrances listed are the justification for what many community development organization do—clarify tenure rights, build consensus and trust, and enhance the adaptive capacity to changing political and project-cycle environments [72, 73]. While similar to the first group with regard to inequalities, this group sees the short-term nature of such cycles (referred to as short termism) as major constraint. Biophysical attributes don’t appear to be of concern, neither does a bridging, polycentric governance body. The landscape specific trial appears to focus on the actionable components of the generic trial. For example, an ‘absence of credible and legitimate spatial planning’ is mitigated by ‘capacity building’ and ‘reaching consensus over goals’ and boundaries (S1 Table). A prominent part of the current development issue cycle relates to community access rights to local resources and therefore many institutions are involved community mapping. In Indonesia, social forestry and the transfer of 12.7 million ha of state forest to local ownership exemplifies this trend [74]. The low ranking of inadequate data on market risks might represent tendency for community development groups to preferentially avoid market-driven approaches to development. The perspectives in this group come from a mix of international researchers, Indonesian researchers and civil servants. The civil servants in this group represent local levels of government, rather than centralized agencies.

#### Group 4. Integrationists

This group sees that the main obstacle to landscape functionality is governance incoherence. Specifically, bureaucratic politics inhibits holistic management (see Sahide, Supratman (71)). They see structural issues in the form of organizational silos leading to incoherent governance from overlapping or conflicting regulations from different actors. The premise for this argument is that if organizations coordinated their efforts, then collaboration between sectors at different levels would mean more effective management. This falls in the domain of political scientists and social-ecological systems theorists who plea for effective polycentric and multi-level governance arrangements [75]. Note that ‘no space for a management institution that integrates….’, is not a problem; in Indonesia there are indeed legislated institutional platforms for integration, such as Forest Management Units that aim to coordinate sectors for integrated management [30, 76]. The lack of clarity of land use categories, user-rights and accountability exemplifies the lack of effectiveness of these institutions due to contested power and unclear authority. Integrationists consist of international and Indonesian researchers.

#### General trial group 5. Democrats

The democrats, a discourse that only emerged in the general trial, are generally unsatisfied with democratic institutions. Lack of transparency and accountability to civil society is primarily a concern of democratic responsiveness [77]. The democrats do not see the rapid change of policies or short termism as a problem, nor election-cycle politics, likely because that is a function of responsive democracy. Their primary concerns for inconsistencies between sectors and poorly harmonized governance structures distinguish them from the social exclusionist discourse. Democratic functionality does not mean inclusively delivered benefits as there are by definition, winners and losers, and as such their primary concerns do not reflect social exclusionary processes. Their concern about inconsistencies, within one administration, means the government does not effectively govern. This discourse suggests that democratic representation by governing bodies will allow for landscape interventions to be allocated in ways that satisfy place-based needs. The democrat discourse came from Indonesians in academia.

#### Specific trial group 6. Neoliberals

The neoliberals see landscape sustainability being constrained by corruption and unpredictable regulatory environments. Markets and trade don’t inhibit landscape functionality, rather the influence of markets and trade should benefit from trustworthy trade and regulatory agreements. The pressure on land and resources guiding government priorities is the major constraint, but rather than regulations and enforcements needing to increase landscape functionality per se, predictability in the regulatory environment is highlighted. Roads and their enabling characteristics for market access and niches are of little concern, either because they are seen as already existing or are public goods to be encouraged. The limits to functional landscapes are therefore related to excessive intervention at the top. The neoliberal perspectives came primarily from Indonesians, comprised of a mix of civil servants, academics and researchers.

### Consensus

For the landscape specific Q-sort trial, there was consensus among all participants over three statements (Table 2). Overall, a constraining element was related to democratic governance. The participants agree that for landscapes to function, especially when thinking about the local contexts of their own landscapes, transparency and accountability to civil society are major hindrances to landscape functionality. An overall lower constraint on landscapes, with local context in mind, was the transition of unconnected poor people to active participation in socio-political economies via connectivity to the outside world. This may represent perceptions that there are no more strictly subsistence livelihoods, or that people are already connected through their social ties beyond the local confines of their livelihoods. Another possible argument is that people shouldn’t have to transition from a subsistence focus to have active participation in the landscape, that landscapes should be inclusive of people whether they lead subsistence-based livelihoods or not. Surprisingly, participants agreed that overlapping and contradictory laws sit neutrally for landscape functionality. This seemingly contradicts what many scholars point out as being fundamentally problematic for Indonesia’s state capability: a complex and ambiguous legal framework [78–80].

**Table 2:** Consensus statements from the landscape specific trial.

	No	Statement	Score
Consensus statements (do not distinguish between any factors)	8	Lack of accountability to civil society, opaque decision making, lack of transparency	2
	14	Slow transition from subsistence focus to active participation in wider landscapes	-2
	22	Overlapping partly contradictory laws with loopholes and lack of grievance procedures	0

We conceive all six discourses as different vantage points of a thematically similar constraint—poor governance. Considering the epistemological and ontological differences among diverse practitioners and academics, one might have assumed that discourses might have aggregated around different domains in the natural and social sciences. Instead, the narratives are all based on different politically situated vantage points of how institutions govern and influence socio-economic development outcomes.

## Discussion

At the beginning of this paper we suggested that the broad range of understandings of a landscape approach implies that implementers are likely to diverge in their perspectives as to what the obstacles are for landscape functionality. To a degree, our results suggest otherwise. A governance leitmotif runs through the overall results and discourses. This suggests that of the many applications and contexts in which they are used, the motivation behind landscape approach implementation is perceived ubiquitous governance failures. However, the overall differences between ranks for ‘generic’ Indonesian landscapes and ‘specific’ landscapes represents fine-tuning of generic perceptions to local contexts.

### Local contexts

Four discourses were similar between the generic and specific trial, with minor but noteworthy differences. Every discourse contains statements that are actionable at the landscape scale, and indeed landscape approach efforts have tried to address them. In addition, the discourses contain exogenous issues that originate at national and/or global scale and require coping mechanisms rather than efforts to modify underlying causes. Although four discourse titles remained the same for the generic and specific trial, the distinguishing statements changed in ways that appear to distil problems into actionable focus items. We see that the general trial discourses favoured statements that are more problem-definition based, and items actionable by organizations are more prominent on the specific landscape trial. For example: in the” seeing like a community” group generic landscape trial, statements such as ‘unequal bargaining power’, ‘absence of credible planning’, and short termism were deemed to be most problematic. However, the corresponding discourse in the specific landscape trial highlighted what many NGOs working in community development do to address these challenges, such as capacity building, consensus building, and trust building [72, 81]. This highlights how participants mentally adjusted from generic problem framing to actual practitioner activities on the ground when moving from generic landscape issues to local contexts.

Statement 23, “increased pressure on land and resources leads to government priorities for economic growth over environmental integrity”, appears in the list of main constraints for the landscape specific trial, and is a more environmentally focused statement than any main constraint in the general trial. The evolutionary origin of landscape approaches is associated with more strictly environmental conceptions of ‘ecosystem approaches’ [23, 82]. But, considering the iterations and evolution of integrated approaches to reconciling conservation and development, it is logical that previous lessons learned have steered conversations toward how governance obstructs management of social-ecological systems [30, 75, 83]. The perception that local demands and priorities collide and contrast with global environmental concerns is shared by others [84], and this is where landscape management strategies must mediate solutions. Management coalitions are needed such that the focal point of landscape governance moves further from simplistic global discourses such as climate change and biodiversity towards a more complex and nuanced approach that responds to the realities of all landscape equity holders and their demands on the landscapes [59].

Other statements that increased in rank of constraints when considering specific landscapes, were; (18) the rapid change of regulations, (30) limited local human capacity, and (10) inaccessible data on economic risks. Scores for the inadequacy of democratic institutions, overlapping laws and corruption became less constraining. This may represent the personal experiences people have with leaders and decision-makers in their own places. The motivation to set aside issues of corruption might displace generic ideological principles when project implementation depends on working with local stakeholders and their pressing needs. Focusing on actionable ways to build civil society seems to be more attractive than tackling institutional failures head-on via rule of law when governments are often the arbiters of legality and have vested interests in maintaining the status quo.

Short termism is only identified as a major constraint by those belonging to the community development discourse. Previous critiques in the scholarly community management discourse identify short termism as a major obstacle [85]. Short termism may be inherent to development, as institutions are entwined with democratic election cycles and the associated donor project-cycles, but might be more problematic now due to “whack-a-mole” policy reactions emerging from rising populism [84]. Global pressures are emerging from populist ‘issue-cycles’ [27], some of which are propelled by policy elites, who have little knowledge of the concerns of communities struggling to survive in the face of economic disadvantage [86].

### Advancing landscape narratives

Critics of landscape approaches claim they are being used to de-politicize the problems apparent in social-ecological systems and entrench neoliberal exclusionary development [22, 23]. In the emergent inadequate governance narrative of our study, the largest discourse group, the social exclusionists, share similar concerns. They see inequitable and exclusive development outcomes as the biggest hindrances to landscape functionality, in the context of sustainability. The landscape approach experts and implementers that comprise that discourse group are not de-politicizing landscapes, rather people and their political institutions are prominent in their problem-framing of landscape approaches. As such, the largest discourse group, the social exclusionists, see problems similarly to how critical development theorists describe problems, such as the exclusionary ‘accumulation by dispossession’ mode of neoliberal development [87–89]. It is clear that landscape approach academics and practitioners in our study are concerned about the inequitable outcomes of current neoliberal modes of development.

But, one discourse group emerged with neoliberal characteristics, comprised of primarily Indonesian nationals who see legitimate needs for a predictable regulatory environment that stimulates economic development through competitive markets and infrastructure connectivity. This mode of development is often criticized in the scholarly literature [47, 90]. Those critiques of development outcomes in Indonesia often come from western scholars who have relatively less at stake in Indonesia’s national development processes [42]. Numerous Indonesian scholars have perceived the value of industrial cropping systems, such as oil palm, differently to critical human geographers of the west [47, 91, 92]. We see value in a tool like Q-methodology in exposing the varying views for better collaborative problem-framing. If our Q-methodology was done with more local forest dwellers it would have likely changed the results. We were not implementing change and did not have stakes in the local landscape development processes. But we are suggesting that if implementing agencies were to intervene in development processes in landscapes, they should account for these views transparently, with a relatively objective tool like the one explored in this paper. Management coalitions, which are described as crucial to the effectiveness of landscape approaches, must not overlook or discount those with different perceptions, especially locally, when trying to advance inclusive development or achieve conservation and development wins [59].

In our study, Indonesians represented actors implementing management decisions, interacting frequently with stakeholders across scales and with local communities. From our results, they hold a wide range of political viewpoints on the major constraints of landscape functionality. The wide range of views show that landscape interventions are subject to multiple knowledge systems, requiring different approaches to building consensus on moving forward. *Mushawara* (community meetings and discussions) are central to Indonesian conflict resolution and collective decision-making processes. Q enables both external people and locals engaging in landscape level *Mushawara* processes to transparently reflect on the differences in perspectives and engage explicitly with opinions that they might deem inappropriate or unexpected [61]. In Indonesia, inclusive *Mushawara* processes are indispensable for reaching consensus over landscape goals and the strategies taken to reach them.

All participants, regardless of their associated discourse, made it clear that reaching consensus among all stakeholders must be a priority, and that it must take place in a forum of mutual-understanding and respect. Coordination among landscape approach implementers will be easier if common concerns are the entry points for their activities. We think analyzing perceptions of landscape implementers and stakeholders with tools such as the Q-methodology adds transparency and helps make theory of change assumptions more rigorously explicit. [2] contend that scenario modeling [93] should be used to make landscape theories of change assumptions explicit. First, landscape approach implementers must clarify points of consensus and divergence among landscape stakeholders. Then they might make progress towards finding the overlaps and differences in their knowledge systems for finding common-concern entry points. And while the primary concerns—corruption, transparency and accountability—are not easily dealt with by landscape level initiatives, they must be part of the main strategic intents for any landscape-scale theory of change otherwise, interventions risk being displacement activities [94]

The challenges of social-ecological systems are complex and often stem from poorly coordinated decisions, where different elements of society frame problems in terms of their own needs and aspirations, leading to unsatisfactory, and often conflicting, zero sum outcomes [7, 30, 31]. Underlying this, is that knowledge is often contested between multiple actors in complex landscapes [27, 28]. In their study on the importance of perceptions on natural resource outcomes, Howe, Corbera [95] demonstrate that actors’ perceptions underpin their policy and management positions, and that policy and management is more likely to fail if their positions mask conflicting values. Landscape stakeholders have a shared responsibility to retain the multi-functionality of landscapes to service future generations and science must contribute to the knowledge, capacity, and motivation for them to do so [62, 63]. If implementers of landscape approaches are going to succeed in achieving their goals, they must come to grips with the actors and discourses at multiple scales; problem framing must be rigorous and collaborative [29]. Recognizing and addressing the diversity of perceptions and discourses of people in a landscape allows for landscape management coalitions to collaboratively problem frame. This should help avoid decisions that do not reflect the values and perceptions of stakeholders in the landscapes that may otherwise provoke conflict or delay success in achieving landscape sustainability [34, 84].

The richness of concourse (Fig 3) around the landscape approach and its prospects for sustainability confirms some conceptual ‘capaciousness’ [18]. The range of terms and concepts enables divergent vantage points in pluralistic societies like Indonesia and in transdisciplinary approaches to problem-driven sustainability science. But we find that landscape approach is not a singular ‘management ethic’ [18]. Rather, political perspectives exist along a spectrum of ethically-bound ‘logics of appropriateness’ [96] for how landscapes should be governed. And while the overall consensus is that corruption, transparency and accountability are seen as the ultimate obstacles, poor governance is encapsulated by a variety of discourses and viewpoints within the landscape approach community. Understanding and making the various vantage points transparent helps landscape approach practitioners to harmonize their efforts with local conceptions of the problems [84].

## Conclusion

To achieve sustainability, landscape approach implementers must understand the comprehensive range of narratives of the problems that they aim to solve. “Policy emerges in a complex process where opinions and concepts matter at least as much as objective evidence, if the latter exists at all” [52]. In this paper we provide evidence that a diverse group of landscape practitioners and researchers have common concerns- that poor governance constrains landscape functionality in Indonesia. The evidence also shows that there is variation in the discourse, depending on the values that underpin one’s political vantage point. Landscape approach implementers must grapple with divergent political vantage points when striving for consensus on the theories of change for landscape development trajectories. As landscape approaches to achieving sustainable development become more prominent in Indonesia and among international agencies to achieve sustainable development, researchers and practitioners must focus on the key obstacles if they want to achieve impact. The results of our discourse analysis show that there are numerous angles from which landscape sustainability is seen to be obstructed by poor governance. We identified six discourse groups among our participants: (1) social exclusionists, (2) state view, (3) community view, (4) integrationists, (5) democrats, and (6) neoliberals. Overall, corruption, transparency and accountability are perceived as the major constraints on landscape functionality. If landscape approach implementers do not address governance issues of major concern and grapple with their own political differences, then interventions risk being displacement activities [94]. Theories of change for landscape approach initiatives must incorporate strategies to account for political stances among landscape stakeholders and rectify governance failures. Only then will sustainability be within sight.

## Supporting information

S1 Table Discourse factors and their defining and distinguishing statements for the general and specific trial. The top 3 'most agree' are landscape constraints, bottom 3 'least agree' are classified least important. Any statement scoring a|3| that was flagged as distinguishing (D = P < .05 and D* = P < .01) is included to add richness to the defining characteristics of the discourse. General trial results on left compared to landscape specific trial results on right. The same discourses arose and are labelled in bold along the left vertical axis. At the bottom are two diverging discourses between the generic and specific trial. Underlines highlight thematic traits defining the discourse type.
(DOCX)

S2 Table Discourse analysis for the general trial. Statements ranked 'most agree' to 'least agree' for each factor. Each factor represents a discourse type. Z-scores determine statement rankings and are the squared differences among from the P-set community flagged for each factor.
(DOCX)

S3 Table Discourse analysis for the landscape specific trial. Statements ranked 'most agree' to 'least agree' for each factor. Each factor represents a discourse type. Z-scores determine statement rankings and are the squared differences among from the P-set community flagged for each factor.
(DOCX)
