# Supplementary material for: Discourses mapped by Q-method show governance constraints motivate landscape approaches in Indonesia
Source: PLoS One. 2019 Jan 31;14(1):e0211221. doi: 10.1371/journal.pone.0211221 (PMC6354971; doi:10.1371/journal.pone.0211221)
Supplement: S1 Table — The top 3 'most agree' are landscape constraints, bottom 3 'least agree' are classified least important. Any statement scoring a|3| that was flagged as distinguishing (D = P < .05 and D* = P < .01) is included to add richness to the defining characteristics of the discourse. General trial results on left compared to landscape specific trial results on right. The same discourses arose and are labelled in bold along the left vertical axis. At the bottom are two diverging discourses between the generic and specific trial. Underlines highlight thematic traits defining the discourse type. (DOCX) [file pone.0211221.s001.docx]

| **General Trial** | | | | | | **Specific Trial** | | | | |
| --- | --- | --- | --- | --- | --- | --- | --- | --- | --- | --- |
| Discourse |  | No. | Statements | Score | Significance |  | No. | Statements | Score | Significance |
| **Social Exclusionists** | Landscape constraints | 27 | Exclusion or underrepresentation of important stakeholders in decision making | 5 | D* | Landscape constraints | 21 | Corruption, personal benefits for those issuing permits | 5 |  |
|  |  | 21 | Corruption, personal benefits for those issuing permits | 4 |  |  | 27 | Exclusion or underrepresentation of important stakeholders in decision making | 4 | D* |
|  |  | 38 | Lack of a common (negotiated, agreed) goal for the landscape as a whole | 4 | D |  | 5 | Inadequate democratic processes and institutions | 4 | D* |
|  |  | 30 | Limited human capacity (knowledge, decision making) within communities and government | 3 | D | Least important | 17 | Spontaneous migrants occupying land | -5 |  |
|  |  | 19 | Land and river degradation by extractive land use activities (e.g. logging, mining, shifting cultivation) | 3 | D* |  | 39 | Rice focus of agricultural policies | -4 |  |
|  | Least important | 39 | Rice focus of agricultural policies | -5 |  |  | 31 | Global climate change, locally changing rainfall patterns | -4 |  |
|  |  | 17 | Spontaneous migrants occupying land | -4 | D* |  | 32 | Uniform government policies while landscapes are diverse in context | -3 | D* |
|  |  | 31 | Global climate change, locally changing rainfall patterns | -4 |  |  | 18 | Regulations change too quickly to be fully applied | -3 | D* |
|  |  | 14 | Slow transition from subsistence focus to active participation in wider landscapes | -3 | D* |  |  |  |  |  |
|  |  |  |  |  |  |  |  |  |  |  |
| **State View** | Landscape constraints | 35 | De- and re-centralization of resource use rights to local government, with benefit sharing between national, provincial, district and village scale | 5 |  | Landscape Constraints | 7 | Absence of effective leadership | 5 |  |
|  |  | 12 | Weak enforcement of existing regulations, poor monitoring of actual change | 4 | D* |  | 21 | Corruption, personal benefits for those issuing permits | 4 |  |
|  |  | 21 | Corruption, personal benefits for those issuing permits | 4 |  |  | 12 | Weak enforcement of existing regulations, poor monitoring of actual change | 4 |  |
|  |  | 18 | Regulations change too quickly to be fully applied | 3 | D* |  | 17 | Spontaneous migrants occupying land | 3 | D* |
|  |  | 7 | Absence of effective leadership | 3 | D* | Least important | 39 | Rice focus of agricultural policies | -5 |  |
|  | Least important | 39 | Rice focus of agricultural policies | -5 |  |  | 36 | Wildlife - farming conflicts (crop raiding) | -4 |  |
|  |  | 10 | Lack of economic data on risk, price fluctuations, market dynamics | -4 | D* |  | 30 | Limited human capacity (knowledge, decision making) within communities and government | -4 |  |
|  |  | 26 | Lack of market premium for products and services from well-managed landscapes | -4 |  |  | 20 | Limited or unsuitable alternatives provided for communities who are banned or prevented from exploiting particular resources | -3 | D* |
|  |  | 22 | Overlapping partly contradictory laws with loopholes and lack of grievance procedures | -3 | D* |  |  |  |  |  |
|  |  |  |  |  |  |  |  |  |  |  |
| **Community View** | Landscape constraints | 4 | Unequal bargaining power, large-scale concessions without local consent | 5 |  | Landscape constraints | 2 | Lack of trust between stakeholders (incl. government, companies, communities) | 5 |  |
|  |  | 1 | Unclear and contested tenure rights, conflicting claims | 4 |  |  | 38 | Lack of a common (negotiated, agreed) goal for the landscape as a whole | 4 | D* |
|  |  | 15 | Absence or credible, legitimate spatial planning | 4 | D |  | 30 | Limited human capacity (knowledge, decision making) within communities and government | 4 |  |
|  |  | 24 | Election cycle politics and focus on quick financial gains | 3 | D | Least important | 39 | Rice focus of agricultural policies | -5 |  |
|  |  | 29 | Reactive policies, projects expecting standard designs and short-term results | 3 | D* |  | 17 | Spontaneous migrants occupying land | -4 |  |
|  | Least important | 31 | Global climate change, locally changing rainfall patterns | -5 | D* |  | 10 | Lack of economic data on risk, price fluctuations, market dynamics | -4 |  |
|  |  | 6 | No space for a management institution that integrates social, environmental and economic aspects of change | -4 | D* |  |  |  |  |  |
|  |  | 33 | Topography constraints to transport, durable roads | -4 |  |  |  |  |  |  |
|  |  |  |  |  |  |  |  |  |  |  |
| **Integrationists** | Landscape constraints | 23 | Increased pressure on land and resources leads to government priorities for economic growth over environmental integrity | 5 | D* | Landscape Constraints | 13 | Lack of clarity on forest categories and associated rules for use, stewardship and accountability | 5 | D* |
|  |  | 9 | Inconsistencies between sectoral policies and misalignment of government structures | 4 |  |  | 35 | De- and re-centralization of resource use rights to local government, with benefit sharing between national, provincial, district and village scale | 4 | D* |
|  |  | 11 | Lack of vertical and horizontal cohesion of government entities | 4 | D* |  | 32 | Uniform government policies while landscapes are diverse in context | 4 |  |
|  | Least important | 24 | Election cycle politics and focus on quick financial gains | -5 |  |  | 11 | Lack of vertical and horizontal cohesion of government entities | 3 | D |
|  |  | 19 | Land and river degradation by extractive land use activities (e.g. logging, mining, shifting cultivation) | -4 |  | Least important | 6 | No space for a management institution that integrates social, environmental and economic aspects of change | -5 | D* |
|  |  | 21 | Corruption, personal benefits for those issuing permits | -4 | D* |  | 7 | Absence of effective leadership | -4 | D* |
|  |  | 8 | Lack of accountability to civil society, opaque decision making, lack of transparency | -3 | D* |  | 31 | Global climate change, locally changing rainfall patterns | -4 |  |
|  |  |  |  |  |  |  | 27 | Exclusion or underrepresentation of important stakeholders in decision making | -3 | D* |
|  |  |  |  |  |  |  |  |  |  |  |
| **Divergent Discourses between general trial and specific trial** | | | | | | | | | | |
| **Democrats** | | | | | | **Neoliberal** | | | | |
| Landscape constraints | | 8 | Lack of accountability to civil society, opaque decision making, lack of transparency | 5 | D* | Landscape constraints | 23 | Increased pressure on land and resources leads to government priorities for economic growth over environmental integrity | 5 | D* |
|  |  | 9 | Inconsistencies between sectoral policies and misalignment of government structures | 4 |  |  | 21 | Corruption, personal benefits for those issuing permits | 4 |  |
|  |  | 2 | Lack of trust between stakeholders (incl. government, companies, communities) | 4 | D* |  | 18 | Regulations change too quickly to be fully applied | 4 |  |
| Least important | | 35 | De- and re-centralization of resource use rights to local government, with benefit sharing between national, provincial, district and village scale | -5 | D* | Least important | 33 | Topography contraints to transport, durable roads | -5 | D* |
|  |  | 36 | Wildlife - farming conflicts (crop raiding) | -4 |  |  | 34 | Lack of market access, not being well-positioned in global economy | -4 | D* |
|  |  | 24 | Election cycle politics and focus on quick financial gains | -4 |  |  | 26 | Lack of market premium for products and services from well-managed landscapes | -4 |  |
|  |  | 29 | Reactive policies, projects expecting standard designs and short-term results | -3 | D |  |  |  |  |  |
