# Supplementary material for: Discourses mapped by Q-method show governance constraints motivate landscape approaches in Indonesia
Source: PLoS One. 2019 Jan 31;14(1):e0211221. doi: 10.1371/journal.pone.0211221 (PMC6354971; doi:10.1371/journal.pone.0211221)
Supplement: S2 Table — Statements ranked 'most agree' to 'least agree' for each factor. Each factor represents a discourse type. Z-scores determine statement rankings and are the squared differences among from the P-set community flagged for each factor. (DOCX) [file pone.0211221.s002.docx]

|  | Factor 1 | | Factor 2 | | Factor 3 | | Factor 4 | | Factor 5 | |
| --- | --- | --- | --- | --- | --- | --- | --- | --- | --- | --- |
| Rank | Statement | Z-score | Statement | Z-score | Statement | Z-score | Statement | Z-score | Statement | Z-score |
| 1 | 27 | 2 | 35 | 2.06 | 4 | 1.803 | 23 | 2.435 | 8 | 2.32 |
| 2 | 21 | 1.452 | 12 | 1.896 | 1 | 1.362 | 9 | 2.114 | 9 | 2.122 |
| 3 | 38 | 1.435 | 21 | 1.819 | 15 | 1.33 | 11 | 1.679 | 2 | 1.932 |
| 4 | 30 | 1.337 | 18 | 1.687 | 24 | 1.319 | 35 | 1.284 | 4 | 1.731 |
| 5 | 8 | 1.029 | 7 | 1.619 | 29 | 1.227 | 22 | 1.057 | 1 | 1.408 |
| 6 | 19 | 0.865 | 17 | 0.961 | 37 | 1.123 | 12 | 0.849 | 12 | 0.863 |
| 7 | 7 | 0.846 | 2 | 0.933 | 5 | 1.01 | 4 | 0.756 | 14 | 0.796 |
| 8 | 15 | 0.826 | 41 | 0.847 | 34 | 0.896 | 37 | 0.756 | 3 | 0.745 |
| 9 | 9 | 0.708 | 1 | 0.831 | 8 | 0.876 | 2 | 0.736 | 6 | 0.606 |
| 10 | 2 | 0.637 | 30 | 0.745 | 13 | 0.832 | 36 | 0.736 | 5 | 0.54 |
| 11 | 3 | 0.615 | 25 | 0.696 | 32 | 0.746 | 38 | 0.642 | 18 | 0.529 |
| 12 | 1 | 0.572 | 40 | 0.571 | 22 | 0.689 | 41 | 0.642 | 31 | 0.519 |
| 13 | 12 | 0.544 | 24 | 0.531 | 27 | 0.688 | 14 | 0.622 | 27 | 0.393 |
| 14 | 4 | 0.517 | 8 | 0.434 | 9 | 0.428 | 18 | 0.548 | 7 | 0.339 |
| 15 | 37 | 0.491 | 32 | 0.423 | 26 | 0.393 | 10 | 0.528 | 22 | 0.264 |
| 16 | 11 | 0.49 | 13 | 0.268 | 41 | 0.376 | 1 | 0.415 | 40 | 0.248 |
| 17 | 20 | 0.488 | 29 | 0.138 | 12 | 0.352 | 17 | 0.321 | 10 | 0.139 |
| 18 | 22 | 0.38 | 4 | 0.079 | 18 | 0.274 | 5 | 0.301 | 15 | 0.136 |
| 19 | 5 | 0.357 | 11 | 0.035 | 21 | 0.184 | 26 | 0.301 | 30 | -0.003 |
| 20 | 40 | 0.313 | 27 | -0.057 | 2 | 0.143 | 28 | 0.207 | 34 | -0.008 |
| 21 | 23 | 0.285 | 31 | -0.306 | 35 | 0.119 | 3 | -0.094 | 17 | -0.062 |
| 22 | 6 | 0.116 | 34 | -0.314 | 23 | 0.088 | 13 | -0.094 | 28 | -0.072 |
| 23 | 24 | -0.008 | 15 | -0.374 | 11 | 0.073 | 40 | -0.321 | 33 | -0.075 |
| 24 | 16 | -0.076 | 6 | -0.389 | 14 | -0.001 | 29 | -0.415 | 32 | -0.141 |
| 25 | 32 | -0.126 | 28 | -0.429 | 20 | -0.058 | 25 | -0.435 | 21 | -0.2 |
| 26 | 29 | -0.152 | 14 | -0.431 | 28 | -0.132 | 27 | -0.528 | 41 | -0.219 |
| 27 | 35 | -0.188 | 9 | -0.555 | 7 | -0.2 | 31 | -0.528 | 16 | -0.26 |
| 28 | 13 | -0.194 | 19 | -0.565 | 40 | -0.46 | 32 | -0.528 | 37 | -0.342 |
| 29 | 28 | -0.225 | 3 | -0.571 | 38 | -0.666 | 39 | -0.548 | 20 | -0.462 |
| 30 | 41 | -0.244 | 36 | -0.573 | 10 | -0.684 | 16 | -0.622 | 38 | -0.542 |
| 31 | 25 | -0.805 | 37 | -0.576 | 16 | -0.832 | 34 | -0.642 | 13 | -0.725 |
| 32 | 18 | -0.86 | 5 | -0.581 | 17 | -0.859 | 6 | -0.756 | 25 | -0.796 |
| 33 | 10 | -0.882 | 20 | -0.675 | 3 | -0.884 | 7 | -0.756 | 26 | -0.928 |
| 34 | 26 | -1.006 | 33 | -0.688 | 25 | -0.898 | 15 | -0.83 | 19 | -1.056 |
| 35 | 34 | -1.211 | 23 | -0.952 | 30 | -0.944 | 20 | -1.037 | 11 | -1.114 |
| 36 | 14 | -1.214 | 16 | -1.012 | 19 | -0.966 | 8 | -1.057 | 23 | -1.189 |
| 37 | 33 | -1.523 | 38 | -1.033 | 39 | -1.079 | 30 | -1.264 | 39 | -1.272 |
| 38 | 36 | -1.529 | 22 | -1.094 | 36 | -1.255 | 33 | -1.284 | 29 | -1.389 |
| 39 | 31 | -1.68 | 26 | -1.423 | 33 | -1.521 | 21 | -1.472 | 24 | -1.454 |
| 40 | 17 | -2.018 | 10 | -1.658 | 6 | -2.332 | 19 | -1.812 | 36 | -1.531 |
| 41 | 39 | -2.362 | 39 | -2.316 | 31 | -2.56 | 24 | -1.907 | 35 | -1.793 |
| Cumulative explained variance: 54% | | | | | | | | | | |
