# Supplementary material for: Discourses mapped by Q-method show governance constraints motivate landscape approaches in Indonesia
Source: PLoS One. 2019 Jan 31;14(1):e0211221. doi: 10.1371/journal.pone.0211221 (PMC6354971; doi:10.1371/journal.pone.0211221)
Supplement: S3 Table — Statements ranked 'most agree' to 'least agree' for each factor. Each factor represents a discourse type. Z-scores determine statement rankings and are the squared differences among from the P-set community flagged for each factor. (DOCX) [file pone.0211221.s003.docx]

|  | Factor 1 | | Factor 2 | | Factor 3 | | Factor 4 | | Factor 5 | |
| --- | --- | --- | --- | --- | --- | --- | --- | --- | --- | --- |
| Rank | Statement | Z-score | Statement | Z-score | Statement | Z-score | Statement | Z-score | Statement | Z-score |
| 1 | 21 | 2.099 | 23 | 2.103 | 2 | 1.667 | 7 | 2.171 | 13 | 1.78 |
| 2 | 27 | 1.859 | 21 | 1.408 | 38 | 1.575 | 21 | 2.092 | 35 | 1.665 |
| 3 | 5 | 1.758 | 18 | 1.358 | 30 | 1.557 | 12 | 1.427 | 32 | 1.295 |
| 4 | 4 | 1.597 | 12 | 1.314 | 7 | 1.503 | 2 | 1.33 | 4 | 1.288 |
| 5 | 1 | 1.509 | 32 | 1.046 | 9 | 1.476 | 32 | 1.104 | 9 | 1.246 |
| 6 | 2 | 0.93 | 40 | 0.992 | 4 | 1.026 | 17 | 1.025 | 11 | 1.043 |
| 7 | 37 | 0.927 | 27 | 0.964 | 1 | 0.954 | 25 | 0.984 | 26 | 1.01 |
| 8 | 24 | 0.924 | 30 | 0.956 | 3 | 0.822 | 31 | 0.911 | 1 | 0.977 |
| 9 | 8 | 0.892 | 9 | 0.919 | 18 | 0.762 | 8 | 0.879 | 8 | 0.773 |
| 10 | 25 | 0.642 | 24 | 0.783 | 27 | 0.739 | 18 | 0.874 | 18 | 0.665 |
| 11 | 7 | 0.554 | 37 | 0.599 | 41 | 0.726 | 24 | 0.854 | 34 | 0.636 |
| 12 | 12 | 0.511 | 29 | 0.56 | 8 | 0.711 | 35 | 0.619 | 22 | 0.62 |
| 13 | 15 | 0.468 | 8 | 0.514 | 28 | 0.703 | 15 | 0.452 | 15 | 0.557 |
| 14 | 20 | 0.405 | 38 | 0.486 | 12 | 0.678 | 38 | 0.408 | 21 | 0.497 |
| 15 | 23 | 0.359 | 7 | 0.383 | 37 | 0.597 | 29 | 0.381 | 41 | 0.482 |
| 16 | 19 | 0.333 | 20 | 0.377 | 22 | 0.488 | 22 | 0.298 | 20 | 0.419 |
| 17 | 38 | 0.296 | 3 | 0.308 | 40 | 0.481 | 9 | 0.237 | 29 | 0.419 |
| 18 | 22 | 0.097 | 11 | 0.267 | 13 | 0.413 | 11 | 0.137 | 12 | 0.341 |
| 19 | 30 | 0.04 | 1 | 0.257 | 16 | 0.026 | 6 | 0.073 | 23 | 0.23 |
| 20 | 13 | -0.003 | 15 | 0.193 | 19 | -0.037 | 4 | 0.033 | 38 | 0.203 |
| 21 | 3 | -0.083 | 39 | 0.031 | 32 | -0.088 | 1 | 0.018 | 5 | 0.18 |
| 22 | 40 | -0.087 | 35 | 0 | 21 | -0.118 | 13 | -0.069 | 3 | 0.167 |
| 23 | 41 | -0.103 | 10 | -0.075 | 20 | -0.14 | 41 | -0.078 | 37 | 0.167 |
| 24 | 29 | -0.126 | 16 | -0.136 | 11 | -0.209 | 37 | -0.116 | 28 | 0 |
| 25 | 11 | -0.203 | 4 | -0.187 | 6 | -0.226 | 23 | -0.151 | 16 | -0.141 |
| 26 | 9 | -0.364 | 28 | -0.219 | 31 | -0.386 | 27 | -0.297 | 24 | -0.263 |
| 27 | 16 | -0.427 | 22 | -0.231 | 15 | -0.476 | 40 | -0.44 | 2 | -0.318 |
| 28 | 6 | -0.444 | 36 | -0.308 | 23 | -0.502 | 33 | -0.485 | 10 | -0.318 |
| 29 | 10 | -0.502 | 19 | -0.336 | 29 | -0.51 | 28 | -0.632 | 17 | -0.321 |
| 30 | 28 | -0.579 | 13 | -0.386 | 34 | -0.518 | 5 | -0.665 | 40 | -0.659 |
| 31 | 35 | -0.605 | 2 | -0.444 | 33 | -0.655 | 10 | -0.762 | 30 | -0.761 |
| 32 | 34 | -0.66 | 6 | -0.492 | 24 | -0.929 | 34 | -0.836 | 19 | -0.79 |
| 33 | 14 | -0.826 | 5 | -0.827 | 35 | -0.98 | 16 | -0.995 | 14 | -0.839 |
| 34 | 36 | -0.996 | 14 | -0.901 | 5 | -1.038 | 26 | -1.04 | 25 | -1.043 |
| 35 | 26 | -1.159 | 41 | -0.903 | 14 | -1.063 | 3 | -1.045 | 33 | -1.105 |
| 36 | 32 | -1.17 | 17 | -1.058 | 25 | -1.083 | 20 | -1.067 | 27 | -1.219 |
| 37 | 18 | -1.288 | 25 | -1.3 | 26 | -1.092 | 14 | -1.126 | 36 | -1.298 |
| 38 | 33 | -1.303 | 31 | -1.498 | 36 | -1.127 | 19 | -1.131 | 39 | -1.563 |
| 39 | 31 | -1.592 | 26 | -1.721 | 10 | -1.186 | 30 | -1.202 | 31 | -1.78 |
| 40 | 39 | -1.593 | 34 | -2.159 | 17 | -2.156 | 36 | -1.96 | 7 | -2.121 |
| 41 | 17 | -2.085 | 33 | -2.64 | 39 | -2.384 | 39 | -2.209 | 6 | -2.124 |
| Cumulative explained variance: 55% | | | | | | | | | | |
